# Supplementary material for: The Somatic Mutation Paradigm in Congenital Malformations: Hirschsprung Disease as a Model
Source: Int J Mol Sci. 2021 Nov 16;22(22):12354. doi: 10.3390/ijms222212354 (PMC8624421; doi:10.3390/ijms222212354)
Supplement: Supplementary file 1 [file ijms-22-12354-s001.zip › Supplementary tables.pdf]

International Journal of *Molecular Sciences*

MDPI

Supplementary Tables

Supplementary Table S1. Exome sequencing quality information.

Patient	Cell type	reads	Mapped reads	Mapped reads (%)	Mean coverage	20X coverage (n bases)	Overlapping 20X coverage	Variants (n)[#]	Overlapping variants (n)[#]	Concordance rate 20 X (%)
**1**	blood	43692456	42653058	97.6	-	1009526	891280	77521	65576	98.4
**1**	ENCC	45158829	44428963	98.4	-	936993		87810		-
**2**	blood	46663496	45464802	97.4	-	1052613	1005681	82750	73769	99.0
**2**	ENCC	51612528	50248904	97.4	-	1056654		85342		-
**3**	blood	73084700	72360584	99.0	-	2951928	2765133	92565	64458	98.8
**3**	ENCC	71473344	70723194	99.0	-	2887416		95400		-
**4**	fibroblasts	71515650	70770498	99.0	-	2896756	1988647	95491	65780	98.6
**4**	ENCC	68160814	58726936	86.2	-	2068315		109987		-
**5**	blood	72927952	72048920	98.8	-	2917188	2661452	96952	84900	99.6
**5**	ENCC	69489918	68471026	98.5	-	2768779		97171		-

Depicted are the number of reads sequenced and mapped to the hg19 reference genome. Coverage statistics and ENCC-germline concordance rate at 20X coverage represent the starting amount of bases at which somatic mosaicism was evaluated. [#]variants counted with a read depth of five or higher at 20X coverage.

Supplementary Table S3. Germline variants in HSCR disease genes.

Patient	Gene	cDNA	Type	dbSNP	gnomAD exome	gnomAD Genome	CADD	Class
**1**	*TBATA*	c.T666C	Synonymous	rs2254433	0.26	0.30	-	LB
**1**	*VCL*	c.G2388A	Synonymous	rs767809	0.48	0.43	-	LB
**1**	*ZEB2*	c.G3480A	Synonymous	-	.	.		LB
**2**	*GFRA1**	c.A1T	Start site loss	-	.	.	23	VUS
**2**	*ZEB2*	c.G3480A	Synonymous	-	.	.	-	LB
**2**	*DENND3*	c.C1110T	Synonymous	rs2289001	0.34	0.27	-	LB
**4**	*NRG3**	c.C59G	Missense	rs1884282	0.12	0.13	3.3	LB
**4**	*NRG3*	c.A1770G	Synonymous	rs17101196	0.08	0.08	-	LB
**4**	*NRG3*	c.C1986T	Synonymous	rs2295933	0.38	0.38	-	LB
**4**	*EDNRB**	c.G1392A	Missense	-			25.7	VUS
**4**	*ZEB2*	c.G3480A	Synonymous	-	.	.	-	LB
**4**	*DENND3*	c.G3090A	Synonymous	rs1045303	0.34	0.28	-	LB
**5**	*NUP98*	c.G2688A	Synonymous	rs35803045	0.05	0.05	3.8	LB

All variants are heterozygous and detected in both cell types. Variants marked with * are tested and confirmed with Sanger sequencing. Abbreviations - LB: Likely Benign, VUS: Variant of uncertain significance, CADD: Combined Annotation Dependent Depletion (http://cadd.gs.washington.edu/), Population frequency depicted is based on the GnomAD genome sequence value (http://gnomad.broadinstitute.org/).

Supplementary Table S4. Rare CNVs identified.

Patient	Chromosome Region	Event	Length	Cytoband	Probes	Gene Symbols	Notes	Classification
1	chr1:185,109,784-185,132,629	CN Gain	22846	q25.3	41	TRMT1L, SWT1	rare CNV	VUS
1	chr1:41,346,254-41,380,652	CN Gain	34399	p34.2	62	.	rare CNV	Likely Benign
1	chr11:54,820,982-54,849,549	CN Gain	28568	q11	25	.	rare CNV	Likely Benign
1	chr5:98,757,280-98,811,517	CN Loss	54238	q21.1	45	.	rare CNV	Likely Benign
2	chr14:69,924,025-69,983,696	CN Gain	59672	q24.1	84	SLC39A9, BC062762, PLEKHD1	rare CNV	VUS
2	chr17:30,106,771-30,107,696	CN Loss	926	q11.2	5	.	rare CNV	Likely Benign
2	chr8:43,727,152-43,831,881	CN Gain	104730	p11.1	23	.	rare CNV	Likely Benign
2	chrX:5,159,410-5,163,874	Homozygous Copy Loss	4465	p22.32	5	.	rare CNV	Likely Benign
3	chr2:10,664,398-10,914,786	CN Gain	250389	p25.1	70	NOL10, RN7SL832P, ATP6V1C2	rare CNV	VUS
4	chr1:37,629,746-37,692,299	CN Loss	62554	p34.3	7	.	rare CNV	Likely Benign
5	chr6:22,008,230-22,093,109	CN Loss	84880	p22.3	32	CASC15, LINC00340	rare CNV	VUS
5	chrX:33,886,710-34,031,196	Homozygous Copy Loss	144487	p21.1	15	.	rare CNV	Likely Benign

Abbreviations - CN: Copy Number, CNV: Copy Number Variant, VUS: Variant of uncertain significance

Supplementary Table S5. Patients included in this study, their clinical features and complications.

Patient	Age at surgery (days)	Length HSCR	Additional features	Complications post-surgery
1	88	15cm; rectosigmoid	None	No complications
2	132	13cm; rectosigmoid	None	Hirschsprung Associated Enterocolitis
3	194	15cm; rectosigmoid	None	Obstructed defecation requiring rectal flushings
4	105	15cm; rectosigmoid	None	Hirschsprung Associated Enterocolitis
5	114	10cm; rectosigmoid	None	No complications
